# Detoxification Center-Based Sampling Missed a Subgroup of Higher Risk Drug Users, a Case from Guangdong, China

**DOI:** 10.1371/journal.pone.0035189

**Published:** 2012-04-17

**Authors:** Peng Lin, Man Wang, Yan Li, Qiaoli Zhang, Fang Yang, Jinkou Zhao

**Affiliations:** 1 Center for Disease Control and Prevention of Guangdong Province, Guangzhou, China; 2 Zhongshan Municipal Center for Disease Control and Prevention, Zhongshan, China; 3 Dongguan Municipal Center for Disease Control and Prevention, Dongguan, China; 4 The Global Fund to Fight AIDS, Tuberculosis and Malaria, Geneva, Switzerland; University of Rochester, United States of America

## Abstract

**Background:**

Injection drug use remains among the most important HIV transmission risk in China. Representativeness of drug users sampled from detoxification centers is questionable. A respondent driven sampling survey was conducted to compare the results with those from the detoxification center in the same city.

**Methods:**

In 2008, two independent surveys were conducted in Dongguan, China, one for community-based drug users using respondent driven sampling and the other for drug users in a compulsory detoxification center as routine sentinel surveillance. Demographic and behavioral information were collected using the same structured questionnaire. Intravenous blood samples were collected to measure antibodies to HIV-1, and syphilis.

**Results:**

Compared to those 400 drug users recruited from the detoxification center, the 303 community-based drug users had higher HIV prevalence (14.7% versus 4.0%, P = 0.04), lower syphilis prevalence (4.7% versus 10.8%, P = 0.07), higher proportion of injection drug use (83.9% versus 60.2%, P = 0.01) and syringe sharing (47.8% versus 36.3%, P = 0.10), more likely to be separated (12.4% versus 3.8%, P = 0.01) and being migrants from Guangxi province (31.4% versus 18.0%, P = 0.09), more engaging in commercial sex (64.4% versus 52.5%, P = 0.04). HIV prevalence and rate of syringe sharing were consistently higher among drug users from Guangxi.

**Conclusions:**

Detoxification center-based sampling missed a subgroup with higher HIV prevalence and higher rate of injection drug use. While detoxification center-based sampled can be used to monitor the trend of HIV prevalence and risk behaviors over time, periodic community-based sampling is still necessary to avoid possible systematic error in detoxification center-based samples.

## Introduction

China had the largest estimated number of injection drug users (IDUs) worldwide, with the mid-point estimate of 2.35 million (1.8 to 2.9 million) in 2005 [1]. Injection drug use (IDU) has been the most important risk factor for HIV spread in China since the epidemic was identified in Yunnan in 1989 [2]. By the end of 2009, IDU accounted for 32.2% the total HIV cases in China [3]. Guangdong is a coastal province with its HIV epidemic primarily driven by IDU [4]. Guangdong lies on a major route of heroin trade from the Myanmar border through Yunnan and Guangxi towards the north of China.

Started in the 1990s, China official drug policy advocated strictly punitive approaches (“Yan Da”). The central government enacted the “Regulations on Prohibition against Narcotics”. Regular illicit drug users and those with drug dependencies would be detained in detoxification centers for periods from 1 to 6 months [5].

To obtain HIV serological and behavioral data among drug users, China CDC established and gradually improved its national HIV sentinel surveillance system from 1995 onwards, with detoxification centers as the sentinel sites [6]. In Guangdong, there are 40 sentinel sites for drug users, exclusively detoxification center-based [4], [7]. Only a portion of drug users are registered, of whom some are arrested and detained in compulsory detoxification centers [8]. Data from sentinel surveillance may not be generalizable to the drug using population at large. Realizing the limitation of the national sentinel surveillance, several community based studies have conducted in some of the drug hardest-hit regions in China, all of which have been based on convenience sampling [9]–[13].

Recently, a modified form of chain-referral sampling called respondent-driven sampling, has been developed and applied for surveying hidden populations such as illicit drug users [14]–[16]. RDS asks participants to recruit their peers, offers a limited number of referral coupons and a financial reward for each recruitment. According to the theory, RDS allow asymptotically unbiased population estimates and inferences [14], [16].

Adjacent to Shenzhen, Dongguan, a city located in the Pearl River Delta region of Guangdong, was selected for this study. The Pearl River Delta region has experienced explosive economic development over the last 20 years, attracted many migrants from other areas of China to find jobs [17]. Dongguan hosts a population of 7.55 million in total and 5.87 million migrants [18]. The HIV epidemic of Dongguan is driven by IDU as other regions of the Pearl River Delta region [4], [18]–[19]. There is only one compulsory detoxification center in Dongguan, which became a sentinel site since 2000. Fluctuates in HIV prevalence from this sentinel site, 2.89% in 2002, 6.24% in 2004, 3.75% in 2006, 4.74% in 2007 [19]–[20], raised the question if detoxification center-based data can represent the real situation in the city. Therefore the present study was conducted to compare the prevalence of HIV, syphilis and risk behaviors between drug users recruited in the community using RDS and those enrolled into sentinel surveillance from the detoxification center.

## Methods

### Study participants and procedure*s*

During the surveillance period defined by the national HIV sentinel surveillance protocol [21], drug users, detained at Dongguan Compulsory Detoxification Center during the month prior to the commencement of sentinel surveillance, were enrolled into sentinel surveillance. Short questionnaire about basic demographic and risk behavioral information and serological test of HIV and syphilis were administered by the health staff according to the national standardized protocol [6], [21]. Each participant was assured of his/her confidentiality in the study and provided oral informed consent before the interview.

The community based sample was recruited using RDS. The eligibility criteria included, self-reported drug use in the previous 6 months; at least 18 years old; residing in Dongguan for at least 3 months; not too intoxicated or otherwise incapacitated to comprehend and provide informed consent; no duplicate participation. For safety consideration, the interview site was a private room in a local health examination clinic. Through methadone maintenance treatment (MMT) clinics, we got contact with local drug users and distributed 20 coupons, with information of the interview site and a valid period of 14 days, to those who would like to participate. Six subjects returned, and were screened for eligibility and provided informed consent. These six subjects were identified as ‘seeds’. Face-to-face interview was administered by the same group of health staff. Intravenous blood samples were collected for the measurement of HIV and syphilis antibodies. A 50 Chinese Yuan (nearly equal to 7.5$) “primary incentive” was paid as compensation for his/her time in completing the interview. Each seed was provided with 5 recruitment coupons and instructed to give them to drug users they knew. A unique number was assigned to each coupon, which enables the linkage between recruiter and recruits. A 20 Chinese Yuan (nearly equal to 3$) was provided to the recruiter for each subject he or she recruited as a secondary incentive. Peer-recruited respondents returning these coupons were then screened for eligibility, interviewed and provided with the primary incentive and 5 coupons. The seventh seed was added at the third week to facilitate recruitment. Age, sex and injection drug use were used to monitor the equilibrium by waves of the recruitment. Based on pre-existing knowledge we estimated that 5% of the community-based drug users are infected with HIV. Having a design effect of 2 and a stand error of no greater than 0.02, 238 drug users would be enough to measure the HIV prevalence [22].

In both detoxification center-based surveillance and community-based survey, recruitment didn't intentionally exclude those subjects who participated in the previous rounds of surveillance or knew their HIV positive status.

### Behavioral measures

The interviews in the compulsory detoxification center collected information on demographics and questions related to drug use and commercial sexual risk behaviors according to the national standardized protocol. The demographic variables included age, gender, place of residency (Hukou), marital status, and education. Drug use risk behaviors included mainly used drug (heroin, opium, other), injection drug use, duration of injecting drug use, syringe sharing. Commercial sexual risk behaviors included buying sex, selling sex, condom use during the last commercial sex episode.

The questionnaire applied in the community-based survey was developed by a group of national and provincial experts in this area, and revised after formative interview with 15 local community-based drug users. All items in the national surveillance questionnaire were included in the questionnaire for community-based survey.

### Laboratory measures

In both of the surveys, blood specimens were screened for HIV antibody by enzyme-linked immunosorbent assay (ELISA) (Beijing King Hawk Pharmaceutical Co., China). A positive ELISA specimen was confirmed by an HIV-1/2 Western Blot immune assay (HIV-1/2 Blot 2.2 WB, Genelabs Diagnositics, Singapore). Samples Western Blot confirmed positive were considered HIV-positive. Syphilis serostatus was determined by a positive syphilis toluidine red untreated serum test (TRUST) test (Shanghai Rongsheng Biotechnologies Co., China).

### Data analysis

Both questionnaires in the community-based survey and sentinel surveillance were double-entered with Epi Data software. The community-based data were statistically adjusted respondent driven sampling analysis (RDSAT) version 6.0 (available free at: www.respondentdrivensampling.org) to produce point prevalence estimates and 95% confidence intervals (CI) for serological, demographic, and HIV risk related characteristics for variables [15], [16]. Analysis of data collected from the sample recruited from detoxification center was performed using SPSS13.0. Statistical difference was defined if 95% confidence intervals were not overlapping for the same variable in both samples. For demographic variables with statistical difference identified, additional analyses were performed to compare major behavioral and serologic variables among subgroups.

## Results

From April to June 2008, 400 drug users were recruited at Dongguan Compulsory Detoxification Center, and completed the questionnaire interview and serological tests.

In the community-based survey, between July and October 2008, 1,500 recruitment coupons were distributed over 11 waves of recruitment; 301 individuals returned and screened for eligibility. Of the screened participants, 290 were found to be eligible and completed the interview; 287 blood samples were collected. Of those ineligible, 20 were found to be duplicate participants by the study staff and one reported no drug use in the previous 6 months. Equilibrium was reached at wave 8 based on the proportion of age, sex and injection drug use.

### Socio-demographic characteristics

Comparisons of socio-demographic characteristics between drug users enrolled into sentinel surveillance and community-based study are presented in Table 1. The community-based drug users were more likely to be separated, and with Guangxi Hukou, while the sentinel surveillance recruited more subjects who were married, and with Hukou for other areas than Guangdong province.

**Table 1:** Social demographic characteristics among drug users from the detoxification center and community.

Variable	Detoxification center (n = 400)		Community (n = 303)		
	%	95% CI	Crude%	Adjusted%	Adjusted 95% *CI*
Gender					
Male	91.2	88.4–94.0	95.0	94.8	90.8–98.0
Female	8.8	6.0–11.6	5.0	5.2	2.0–9.2
Age(years)					
≤25	27.2	22.8–31.6	22.8	25.4	17.8–33.1
>25	72.8	68.4–77.2	77.2	74.6	66.9–82.2
Education, years					
<6	36.0	31.3–40.7	32.3	34.6	27.4–42.1
6–9	56.5	51.6–61.4	59.4	56.2	48.5–63.4
>9	7.5	4.9–10.1	8.3	9.2	4.7–14.9
Marital status					
single	56.5	51.6–61.4	55.8	57.1	49.7–65.6
married	39.8	35.0–44.6	32.0	30.5	22.8–37.0
separated	3.8	1.9–5.7	12.2	12.4	7.5–18.0*
Residency					
Local	19.0	15.2–22.8	32.7	28.3	17.9–40.7
Guangxi	18.0	14.2–21.8	22.4	31.4	20.5–42.8
Other	63.0	58.3–67.7	44.9	40.3	28.2–52.5*

IDU injection drug use. *CI* confidence interval.

* Denotes significance at alpha value of 0.05.

### Drug use and sexual behaviors

As shown in Table 2, compared with drug users enrolled into sentinel surveillance, the community-based drug users were more likely to be IDUs, engage in commercial sex; during last commercial sex episode, they reported a higher proportion of condom use; they might have slightly longer duration of IDU.

**Table 2:** Drug use and sexual behaviors among drug users from the detoxification center and community.

Variable	Detoxification center (n = 400)		Community (n = 303)		
	%	95% CI	Crude%	Adjusted%	Adjusted 95% *CI*
Used heroin					
Yes	95.5	93.5–97.5	98.0	96.5	92.9–99.4
No	4.5	2.5–6.5	2.0	3.5	0.6–7.1
IDU					
Yes	60.2	55.4–65.0	85.1	83.9	77.7–89.6*
No	39.8	35.0–44.6	14.9	16.1	10.4–22.3*
Duration of IDU(years)**					
<2	42.1	37.3–46.9	26.7	32.1	23.4–39.3
2–4	22.1	18.0–26.2	31.0	30.2	22.6–37.8
≥5	35.8	31.1–40.5	42.2	37.2	29.7–47.7
Needle sharing**					
Yes	36.3	31.6–41.0	51.6	47.8	38.9–57.6
No	63.7	59.0–68.4	48.4	52.2	42.4–61.6
Engaging in commercial sex					
Yes	52.5	47.6–57.4	66.7	64.4	57.7–72.7*
No	47.5	42.6–52.4	33.3	35.6	27.3–42.3*
Condom use during last commercial sex episode#					
Yes	29.2	24.7–33.7	46.0	42.3	34.2–52.5*
No	70.8	66.3–75.3	54.0	57.7	47.5–65.8*

IDU: injection drug use. *CI:* confidence interval.

* Denotes significance at alpha value of 0.05.

** Variable represents characteristic of injection drug users.

# Variable represents characteristic of those engaging in commercial sex.

### Prevalence of HIV and syphilis

All drug users of the sentinel surveillance provided blood specimens, while 3 community-based ones didn't. As shown in Table 3, the estimated syphilis prevalence of the community-based subjects was nearly the same as the crude one, while the estimated HIV prevalence was higher than the crude one. Compared with the sentinel surveillance subjects, the community-based drug users had higher HIV prevalence. The sentinel surveillance recruited more syphilis sero-positive subjects; however, this difference was not statistically significant.

**Table 3:** Prevalence of HIV, syphilis among drug users from the detoxification center and community.

	Detoxification center (n = 400)			Community (n = 300)			
	Positive	%	95%*CI*	Positive	Crude%	Adjusted%	Adjusted 95% *CI*
HIV	16	4.0	2.1–5.9	25	8.3	14.7	6.1–25.4*
Syphilis	43	10.8	7.8–13.8	14	4.7	4.7	1.8–8.2

*CI*: confidence interval.

* Denotes significance at alpha value of 0.05.

A subsample of drug users enrolled at MMT clinics were captured by RDS. The HIV prevalence among drug users who got enrolled at MMT clinics (2.5% or 1/40) was lower than those who didn't (9.2% or 24/260, P = 0.260).

### Behavioral and serologic variables by Hukou

Drug users from Guangxi had higher HIV prevalence and higher rate of needle sharing in both community-based and detoxification center-based subjects. HIV prevalence and needle sharing rate among drug users with Guangxi Hukou were much higher among those recruited in community than those recruited in the detoxification center (Table 4).

**Table 4:** Major behavioral and serologic variables by Hukou among drug users from the detoxification center and community.

	Detoxification center (n = 400)					Community (n = 303)				
	Dongguan%(*n*)	Guangxi%(*n*)	Other%(*n*)	*χ^2^*	*P*	Dongguan%(*n*)	Guangxi%(*n*)	Other%(n)	*χ^2^*	*P*
HIV	2.8( 2)	10.5( 8)	2.4( 6)	10.43	<0.01	4.0( 4)	26.9(18)	2.2( 3)	39.03	<0.01
Syphilis	11.1( 8)	9.2( 7)	11.1( 28)	2.23	0.89	4.0( 4)	6.0( 4)	4.5( 6)	0.35	0.84
Marital status				9.12	0.05				15.54	<0.01
single	47.2(34)	68.4(52)	55.6(140)			49.5(49)	72.1(49)	52.2(71)		
married	51.4(37)	28.9(22)	39.7(100)			41.4(41)	22.1(15)	30.1(41)		
separated	1.4( 1)	2.6( 2)	4.8( 12)			9.1( 9)	5.9( 4)	17.6(24)		
IDU	55.6(40)	56.6(43)	62.5(157)	1.64	0.44	91.9(91)	83.8(57)	80.9(110)	5.64	0.06
Syringe sharing*	27.5(11)	58.1(25)	32.5( 51)	11.20	<0.01	36.3(33)	71.9(41)	53.6(59)	18.18	<0.01

IDU: injection drug use.

* Variable represents characteristic of injection drug users.

## Discussion

The results of this study indicate that, the community-based drug users recruited by RDS had more than 3 times greater HIV prevalence than those enrolled into sentinel surveillance from the detoxification center. The subjects recruited in the community also reported higher HIV-related risk behaviors, being more likely to be IDUs, sharing syringes, and engaging in commercial sex. Further analysis found subjects with Guangxi Hukou is the major subgroup attributable to the difference in HIV prevalence, as subjects with Guangxi Hukou had much higher HIV prevalence and higher rate of need sharing. Our findings are consistent with a community-based study in three areas in Pearl River Delta region in 2002, where HIV prevalence among those community-based drug users was nearly 5 times higher than that among sentinel surveillance subjects [8], [9].

Consistent with our findings, one study in Guizhou province in 2004 also found difference in drug using behavior, being less likely to inject drug in the past 6 months among community recruited subjects than those from detoxification center [23]. As indicated in 2005 national behavior surveillance of drug users in China, drug users recruited in the compulsory detoxification centers had higher HIV prevalence than those recruited in the community [24].

In contrast to higher HIV prevalence, the community-based population had lower syphilis prevalence. Although the community-based drug users reported a higher proportion of engaging in commercial sex, they also reported a higher proportion of condom use during last commercial sex episode. Since the sentinel surveillance only focused on commercial sex behaviors, we were not able to further explore the differences of sex behaviors with regular and casual sex partners. Very low homophily of variables ‘injection drug use’ and ‘syringe sharing’ (data not shown) in the community-based sample indicates differences in these two variables between community-based and detoxification-based samples could possibly be attributable to the difference in the sampling methods.

This study is not without limitations. First the drug users in the detoxification center may under-report the drug using behaviors due to social desirability bias [25], and the nature of being detained while those recruited in the community may report what they are doing due to friendly nature of interview site although social desirability bias may exist as well [24]. For both samples, recall biases exist. Second, the sample size was relatively small for community-based sample. However, the recruitment strictly followed the RDS protocol and equilibrium was reached on key demographic variables. It seems RDS penetrated into the network in the community. Third, the statistical methods for both samples are based on probability sampling, which may not be true for detoxification center based sample. Comparison based on the analytical methods may not the best. However, the sample size of detoxification center based sample was based on the China national HIV sentinel surveillance protocol and was considered to be enough to apply statistical methods for probability samples.

Despite the limitations, this study is among the first in China to critically compare HIV prevalence of the community based drug users with those confined into compulsory detoxification center. Detoxification center-based sample missed a subgroup, which have higher HIV prevalence and higher risk behaviors but lower prevalence of syphilis. Together with findings from other studies in the same region and in other areas in China, it seems detoxification center-based samples may miss important subgroups of drug using population which could influence estimation of HIV prevalence and other characteristics. While samples of drug users consistently recruited from detoxification centers can be used to monitor the trend of serologic and behavioral variables, periodic community-based sampling surveys among drug users in the same geographic areas are still necessary to avoid systematic over- or under-estimation of HIV prevalence or other infection or behavioral information collected in the routine sentinel surveillance.
